# Establishment and verification of prediction model of occult peritoneal metastasis in advanced gastric cancer

**DOI:** 10.1186/s12957-023-03188-2

**Published:** 2023-10-13

**Authors:** Hengfei Gao, Kangkang Ji, Linsen Bao, Hao Chen, Chen Lin, Min Feng, Liang Tao, Meng Wang

**Affiliations:** 1 Department of General Surgery, Nanjing Drum Tower Hospital, Affiliated Hospital of Nanjing University Medical School, Nanjing, China; 2 Medical School of Nanjing University, Nanjing, China; 3 Department of Gastrointestinal, Fuyang People’s Hospital, Fuyang, China; 4 Department of General Surgery, Nanjing Drum Tower Hospital Clinical College of Nanjing Medical University, Nanjing, China

**Keywords:** Gastric cancer, Claudin18.2, CA125, Occult peritoneal metastasis, Nomogram

## Abstract

**Background:**

To investigate the risk factors associated with the development of occult peritoneal metastasis in advanced gastric cancer, and establish and externally validate a nomogram for predicting the occurrence of occult peritoneal metastasis in patients with advanced gastric cancer.

**Methods:**

A total of 111 patients with advanced gastric cancer who underwent laparoscopic exploration or peritoneal lavage cytology examination at the Affiliated Drum Tower Hospital of Nanjing University Medical School from August 2014 to December 2021 were retrospectively analyzed. The patients diagnosed between 2019 and 2021 were assigned to the training set (*n* = 64), while those diagnosed between 2014 and 2016 constituted the external validation set (*n* = 47). In the training set, patients were classified into two groups based on preoperative imaging and postoperative pathological data: the occult peritoneal metastasis group (OPMG) and the peritoneal metastasis negative group (PMNG). In the validation set, patients were classified into the occult peritoneal metastasis group (CY1P0, OPMG) and the peritoneal metastasis negative group (CY0P0, PMNG) based on peritoneal lavage cytology results. A nomogram was constructed using univariate and multivariate analyses. The performance of the nomogram was evaluated using Harrell’s C-index, the area under the receiver operating characteristic curve (AUC), decision curve analysis (DCA), and calibration plots.

**Results:**

This study analyzed 22 potential variables of OPM in 111 gastric cancer patients who underwent laparoscopic exploration or peritoneal lavage cytology examination. Logistic regression analysis results showed that Lauren classification, CLDN18.2 score and CA125 were independent risk factors for OPM in patients with gastric cancer. We developed a simple and easy-to-use prediction nomogram of occult peritoneal metastasis in advanced gastric cancer. This nomogram had an excellent diagnostic performance. The AUC of the bootstrap model in the training set was 0.771 and in the validation set was 0.711. This model showed a good fitting and calibration and positive net benefits in decision curve analysis.

**Conclusion:**

We have developed a prediction nomogram of OPM for gastric cancer. This novel nomogram has the potential to enhance diagnostic accuracy for occult peritoneal metastasis in gastric cancer patients.

## Introduction

Gastric cancer (GC) is the fifth most common type of cancer globally and the fourth leading cause of cancer-related deaths [1]. Due to the asymptomatic nature of early-stage GC, most patients are diagnosed at an advanced stage. Consequently, the prognosis for GC patients remains poor, with a 5-year overall survival rate of only 40–60% in Asia [2, 3].

Peritoneal metastasis (PM) is one of the most common forms of metastasis in gastric cancer. Approximately 53% to 80% of gastric cancer patients experience distant metastasis, and the prognosis is particularly poor [4, 5]. Recent studies have indicated that neoadjuvant chemotherapy administered before surgery has shown significant clinical efficacy and improved overall survival rates in patients with advanced gastric cancer accompanied by peritoneal metastasis [6]. Occult peritoneal metastasis (OPM) refers to the situation where no peritoneal metastasis is detected through imaging examinations before surgery. However, after invasive procedures such as laparotomy or laparoscopy, the presence of peritoneal metastasis is confirmed through pathological examination [7]. This includes cases with positive cytology (CY1) and macroscopic metastatic lesions (P1). Surgical treatment for gastric cancer patients with responsive occult peritoneal metastasis after preoperative chemotherapy is safe and prolongs the survival of P1 and CY1 gastric cancer patients [8, 9]. Therefore, accurate assessment of the presence of peritoneal metastasis is crucial for selecting appropriate patients for neoadjuvant chemotherapy. Computed tomography (CT) is the most common non-invasive method for diagnosing peritoneal metastasis. However, CT imaging features such as large amounts of ascites, significant thickening of the peritoneal wall, peritoneal nodules, and increased opacification of the peritoneum are mostly observed in patients with advanced-stage peritoneal metastasis. CT has high specificity but low sensitivity for detecting peritoneal metastasis [10]. Laparoscopic examination and cytological examination of peritoneal lavage fluid are considered the gold standard for detecting occult peritoneal metastasis. It is recommended that patients at risk of occult peritoneal metastasis undergo these examinations. However, due to the invasive nature of laparoscopic examination and cytological examination of peritoneal lavage fluid, patient acceptance and compliance may be limited.

The tight junction protein family plays a crucial role in epithelial cells, mediating cell–cell adhesion, regulating selective permeability and ion homeostasis, and participating in the regulation of tumor proliferation and differentiation functions [11]. The Claudin 18 subtype 2 (CLDN18.2) of the tight junction protein family is a highly selective marker protein that is expressed exclusively in differentiated gastric mucosal epithelial cells. Its expression is highly limited in normal healthy tissues and is not expressed in undifferentiated gastric stem cells. In normal healthy tissues, CLDN18.2 is buried within the tight junctions of gastric mucosal cells and is largely inaccessible to antibody binding. However, due to malignant transformation and loss of cell polarity, CLDN18.2 gradually becomes exposed on the surface of tumor cells. This unique characteristic has drawn attention to its potential as a therapeutic target for gastric cancer (GC) [12]. Studies have shown that the expression of CLDN18.2 is inversely correlated with the occurrence of peritoneal metastasis (PM) in diffuse-type gastric cancer. Therefore, the expression of CLDN18.2 may play an important role in the diagnosis of occult peritoneal metastasis (OPM) [13].

Previous studies have primarily focused on identifying risk factors for peritoneal metastasis (PM) in gastric cancer. However, research on occult peritoneal metastasis (OPM) in gastric cancer has been limited, with most studies focusing on radiomics research. Currently, there are no widely accepted clinical criteria for OPM. Therefore, the objective of this study is to explore the risk factors for the occurrence of occult peritoneal metastasis in advanced gastric cancer at the clinical and pathological levels. The aim is to establish a nomogram model based on these factors and validate its effectiveness.

## Methods and materials

### Patient section

This is a single-center, retrospective study with the ethical approval of our hospital in accordance with the Declaration of Helsinki. This study retrospectively evaluated the medical data of patients with advanced gastric cancer who underwent laparoscopic examination or cytological examination of peritoneal lavage fluid at the Department of General Surgery, Drum Tower Hospital, affiliated with Nanjing University School of Medicine, from August 2014 to December 2021.The inclusion criteria were as follows: (1) Preoperative gastric endoscopic pathological diagnosis of gastric adenocarcinoma; (2) Preoperative imaging staging indicating advanced gastric cancer (T2-T4a); (3) Patients who underwent either laparoscopic examination or cytological examination of peritoneal lavage fluid; (4) Preoperative CT four-point radiomics analysis: peritoneal opacification score < 2 [14]. The exclusion criteria were as follows: (1) history of or concurrent primary or metastatic malignancies other than gastric cancer; (1) preoperative receipt of neoadjuvant chemotherapy; (2) presence of peritoneal metastasis confirmed by imaging examinations (including CT, MRI, or PET-CT, etc.). At last, a total of 111 patients were enrolled.

### Experimental group

**Fig. 1:**
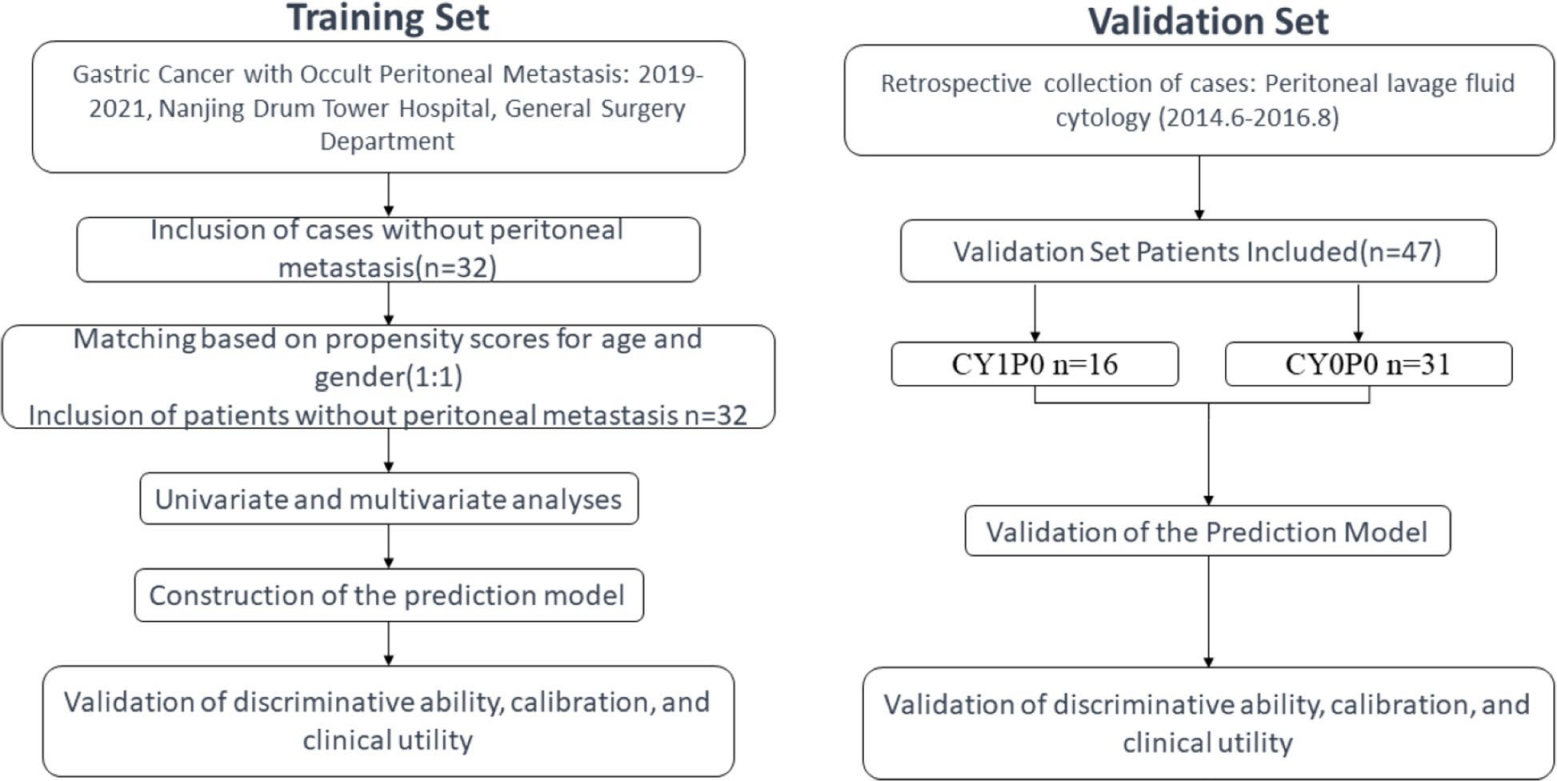
Enrollment flow chart of patient with training set and validation set according to inclusion and exclusion criteria

The training set was divided into two groups based on imaging examinations: the Occult Peritoneal Metastasis Group (OPMG) and the Peritoneal Metastasis Negative Group (PMNG). The validation set was divided based on cytological examination of peritoneal lavage fluid into the cytology-positive group (CY1P0) and the cytology-negative group (CY0P0). The detailed process is presented in Fig. 1.

### Data collection

The data were collected as follows: (1) hematological parameters, including lymphocyte count (L), neutrophil count (N), platelet count (P), neutrophil-to-lymphocyte ratio (NLR), platelet-to-lymphocyte ratio (PLR), serum alpha-fetoprotein (AFP), carcinoembryonic antigen (CEA), carbohydrate antigen 125 (CA125), CA242, CA199, CA724, hemoglobin (Hb), serum albumin (Alb). (2) Pathological data, including tumor location, tumor differentiation type, tumor Lauren classification, preoperative gastric endoscopic Claudin18.2 immunohistochemical IRS score (CLDN18.2 score).

### Diagnostic criteria for occult peritoneal metastasis

**Fig. 2:**
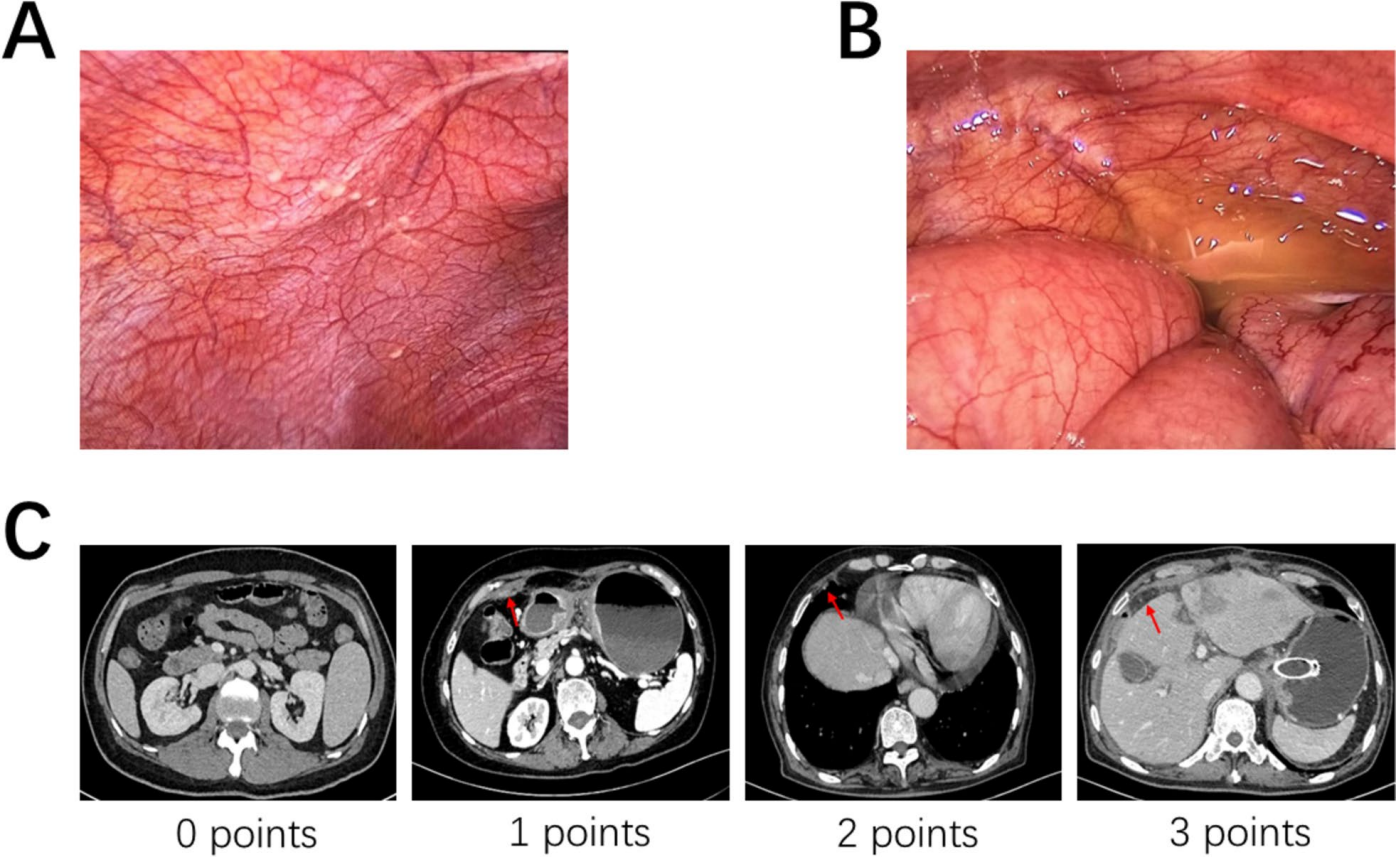
Endoscopic exploration and occult peritoneal metastasis (OPM) score of the peritoneum attached to the organs or tissues. **A** Peritoneal malignant nodules. **B** Malignant ascites in the abdominal cavity. **C** Computed tomography (CT) findings that correspond to the aforementioned criteria. The degree of metastasis is displayed from light to severe

(1) Confirmation of localized peritoneal metastasis by intraoperative rapid pathology or postoperative pathology, including isolated lesions in the greater omentum, lesser omentum, anterior leaf of the transverse mesocolon, pancreatic serosa, and peritoneum near the spleen that are discontinuous with the primary lesion, as well as peritoneal nodules in the upper, middle, and lower abdomen, or detection of tumor cells in ascites or peritoneal lavage fluid cytology (CY1) [15] (Fig. 2A, B). (2) Imaging examination showing a peritoneal opacification score of < 2 [14]. The specific method is as follows: Peritoneal metastasis indicators are observed in the CT images of each section of the patient (Fig. 2C). The scoring criteria are shown in Table 1.

**Table 1:** Definition of the computed tomography (CT) scoring system

Score	Free peritoneum	Peritoneum covering organs or tissues
0	No abnormal sign	No lines displayed
1 (mild)	Slightly and homogeneously increased fat density appearing as S-GGO	Slight thickened line
2 (moderate)	Heterogeneously increased density with patchy or intensive S-GGO	Obviously thickened line with enhancement
3 (severe)	Heterogeneously and obviously increased density with intensive S-GGO, multiple strands, curls sign, or blurred-margined small nodules	Obviously thickened line with enhancement and tiny nodules or a small amount of ascites

*S-GGO* smudge-like ground-glass opacity

### Immunohistochemical scoring of CLDN18.2

**Fig. 3:**
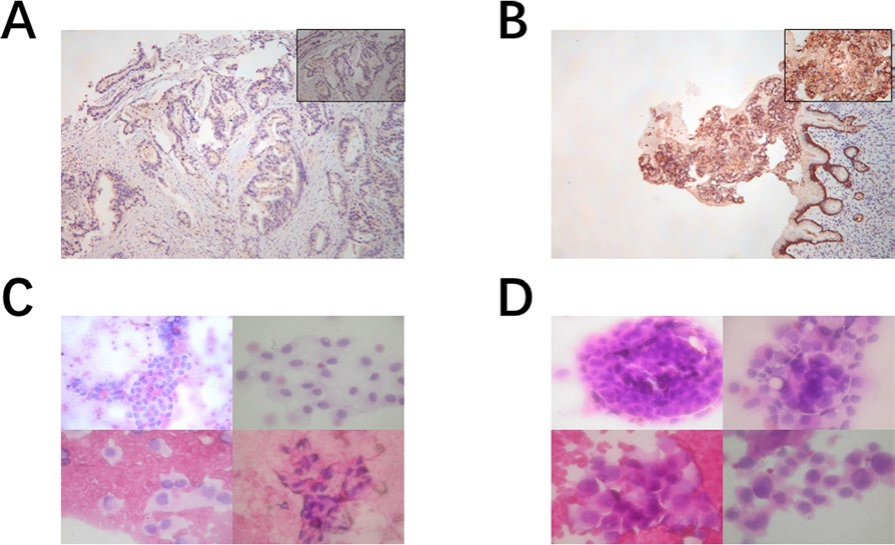
Pathological evaluation: immunohistochemical scoring and cytological examination images. **A** Negative immunohistochemical staining for CLDN18.2. **B** Positive immunohistochemical staining for CLDN18.2. **C** Positive cytological findings in peritoneal lavage fluid. **D** Negative cytological findings in peritoneal lavage fluid

The gastric cancer specimens were obtained from formalin-fixed preoperative gastric biopsy tumor tissue and embedded in paraffin blocks. The samples were heated in a dry oven at 60 °C for at least 1 h, deparaffinized in xylene, dehydrated in 100%, 95%, and 70% ethanol sequentially, and treated with hydrogen peroxide. Antigen retrieval was performed by microwave treatment for 15 min in citrate buffer (pH 6.0). The samples were washed twice on glass slides with 1 × Tris-buffered saline and Tween 20® and blocked with antibody diluent for 10 min. The samples were incubated with primary antibody against CLDN18.2, followed by incubation at 23 °C–25.5 °C in a humidified chamber for 30 min, and then detected with polymer HRP Ms + Rb for 10 min. Visualization of CLDN18.2 was accomplished by incubating with Opal 690 TSA Plus (dilution 1:150) for 10 min, followed by immersion of the fixed samples on glass slides in citrate buffer (pH 6.0) and heat treatment by microwave. Subsequently, the samples were observed and scored by the pathologist under a microscope. According to the IRS scoring criteria, the percentage of positive staining is scored as follows: ≤ 5% = 0, 5% to 25% = 1, 25% to 50% = 2, 50% to 75% = 3, > 75% = 4. The staining intensity is scored as follows: no staining = 0, yellow = 1, light brown = 2, dark brown = 3. The final score is obtained by multiplying the percentage score with the intensity score. Figure 3A, B shows the immunohistochemical images of preoperative gastric biopsy tumor tissue on pathological slides.

### Cytological examination of peritoneal lavage fluid

All the cytological smears of the peritoneal lavage fluid were evaluated by two experienced cytopathologists, and a consensus diagnosis was reached. A cytological positive result was defined as the sample being highly suspected to contain or showing the presence of malignant tumor cells. Conversely, a cytological negative result was indicated when such cells were not found. The microscopic examination results of the cytological evaluation in the cytology-positive group (CY1P0) and cytology-negative group (CY0P0) are shown in Fig. 3C, D, respectively.

### Construction of the nomogram

The demographic characteristics of patients in the training set were compared to those in the validation set to assess for any differences. Based on the patient data in the training set, a univariate analysis was conducted to identify potential risk factors. Normally distributed continuous variables were expressed as mean ± standard deviation, while non-normally distributed continuous variables were presented as median (interquartile range). Independent samples *t* test was used for comparing normally distributed variables between the two groups, and the Mann–Whitney *U* test was used for non-normally distributed variables. Categorical variables were expressed as counts (percentages), and the chi-square test was employed for comparing the two groups. Variables that showed statistical significance (*P* < 0.05) were further analyzed using multivariable logistic regression analysis. The selected independent risk factors were used to construct a nomogram model using R software (version 4.0.2).

### Nomogram performance

The receiver operating characteristic curve (ROC curve) and the area under the curve (AUC) were used to compare and evaluate the discriminative ability of the model and individual predictors. A calibration curve was plotted to assess the calibration of the nomogram model by comparing the predicted probability of OPM occurrence with the actual probability of OPM occurrence. The discriminative ability and calibration of the model were further validated using 1000 bootstrap resamples for internal and external validation. The goodness of fit of the nomogram model was evaluated using the Hosmer–Lemeshow test. The clinical utility of the model was assessed by plotting the decision curve analysis (DCA) curve. Finally, the model was externally validated using patient data from the validation set.

## Results

### Patient characteristics

Among the 111 patients enrolled in this study, they were divided into a training set (*n* = 64) and an external validation set (*n* = 47) based on the year of diagnosis (2019–2021 and 2014–2016, respectively). A comparison of hematological parameters and clinical-pathological parameters between the two groups is presented in Table 2. No significant differences were observed between the training set and validation set.

**Table 2:** Clinicopathologic variables of the training and validation sets

Variable	Training set (*n* = 64)	Validation set (*n* = 47)	*P* value
Age (%)			0.550
≤ 65 years	21 (32.81)	18 (38.30)	
> 65 years	43 (67.19)	29 (61.70)	
Sex (%)			0.402
Male	33 (51.56)	28 (59.57)	
Female	31 (48.44)	19 (40.43)	
BMI (%)			0.984
≤ 18 kg/m^2^	9 (14.06)	7 (14.89)	
18 ~ 25 kg/m^2^	42 (65.62)	31 (65.96)	
≥ 25 kg/m^2^	13 (20.31)	9 (19.15)	
Clinical T stage (%)^a^			0.857
2	9 (14.06)	6 (12.77)	
3	32 (50.00)	26 (55.32)	
4	23 (35.94)	15 (31.91)	
Clinical N stage (%)^a^			0.263
0	17 (26.56)	6 (12.77)	
1	23 (35.94)	24 (51.06)	
2	16 (25.00)	11 (23.40)	
3	8 (12.50)	6 (12.77)	
Neutrophil count (N) × 10^9^/L	3.6 (2.6–4.3)	3.3 (2.6–4.0)	0.654
Lymphocyte count (L) × 10^9^/L	1.6 (1.3–.0)	1.7 (1.3–2.0)	0.876
Platelet count (P) × 10^9^/L	211.0 (157.8–260.8)	188.0 (159.0–258.0)	0.627
NLR	2.2 (1.3–3.0)	1.7 (1.5–2.8)	0.670
PLR	125.0 (90.9–166.7)	111.1 (80.1–166.7)	0.375
Hemoglobin (Hb) g/L	123.5 (104.8–138.0)	132.0 (111.0–142.0)	0.089
Albumin (Alb) g/L	37.9 ± 3.4	38.7 ± 3.4	0.230
AFP ng/L	2.3 (1.7–3.5)	2.7 (1.7–4.1)	0.410
CEA ng/L	1.5 (0.7–3.1)	1.3 (0.6–2.5)	0.303
CA125 ng/L	7.7 (5.1–16.3)	7.9 (4.8–13.2)	0.983
CA242 ng/L	4.1 (3.0–9.8)	3.9 (2.6–9.7)	0.564
CA199 ng/L	8.9 (6.2–27.7)	7.8 (4.0–22.0)	0.305
CA724 ng/L	3.0 (1.4–6.3)	2.1 (1.4–3.8)	0.134
CLDN18.2	2.0 (0.0–8.0)	4.0 (2.0–7.0)	0.395
Tumor location (%)			0.496
Upper	26 (40.6)	15 (31.9)	
Middle	15 (23.4)	10 (21.3)	
Lower	23 (35.9)	22 (46.8)	
Occult peritoneal metastasis (%)			0.094
No	32 (50.0)	31 (66.0)	
Yes	32 (50.0)	16 (34.0)	
Lauren classification (%)			0.057
Diffuse type	31 (48.4)	11 (23.4)	
Intestinal type	18 (28.1)	19 (40.4)	
Mixed type	15 (23.4)	17 (36.2)	
Tumor differentiation type (%)			0.29
Undifferentiated type	38 (59.4)	23 (48.9)	
Mixed type	19 (29.7)	14 (29.8)	
Differentiated type	7 (10.9)	10 (21.3)	

^a^Clinical staging (cTNM) of cancer, *NLR* neutrophil-to-lymphocyte ratio, *PLR* platelet-to-lymphocyte ratio, *CLDN18.2* Claudin18.2 in Gastric Endoscopic Biopsy Specimen Immunohistochemistry Score

### Analysis and development of the nomogram

Logistic univariate and multivariate analyses were performed on the clinical parameters of the training set patients (shown in Table 3). Univariate analysis revealed that tumor location (OR = 2.92, 95%CI 0.91–9.44, *P* = 0.0426), Lauren subtype (OR = 0.21, 95%CI 0.06–0.75, *P* = 0.0163), tumor differentiation (OR = 0.12, 95%CI 0.01–1.11, *P* = 0.0416), CA125 (OR = 1.08, 95%CI 1.01–1.16, *P* = 0.0098), and gastric endoscopic biopsy Claudin 18.2 immunohistochemical score (CLDN18.2 score) (OR = 0.86, 95%CI 0.76–0.97, *P* = 0.0169) were significantly associated with the occurrence of occult peritoneal metastasis in gastric cancer. However, there was no significant association between AFP, CEA, CA242, CA199, CA724, neutrophil count (N), lymphocyte count (L), platelet count (P), neutrophil-to-lymphocyte ratio (NLR), platelet-to-lymphocyte ratio (PLR), hemoglobin (Hb), albumin (Alb), and the occurrence of occult peritoneal metastasis in gastric cancer. Multivariate analysis results showed that Lauren subtype (OR = 0.40, 95%CI 0.18–0.88, *P* = 0.0219), CLDN18.2 score (OR = 0.86, 95%CI 0.75–0.99, *P* = 0.0301), and serum CA125 (OR = 1.11, 95%CI 1.02–1.20,* P* = 0.0123) were identified as independent risk factors for the occurrence of occult peritoneal metastasis in gastric cancer patients.

**Table 3:** Variables associated with OPM according to the logistic regression model

Variable	Univariable analysis		Multivariable analysis	
	OR (95%CI)	*P* value	OR (95%CI)	*P* value
Age (%)	0.65 (0.23–1.87)	0.426		
Sex (%)	1.13 (0.42–3.02)	0.803		
BMI (%)	0.93 (0.17–5.15)	0.937		
Clinical T stage (%)*	1.40 (0.48–4.11)	0.538		
Clinical N stage (%)*	1.12 (0.21–6.05)	0.891		
Neutrophil count (N)	0.76 (0.56, 1.04)	0.0902		
Platelet count (P)	1.00 (0.99, 1.00)	0.5999		
NLR	0.82 (0.61, 1.09)	0.1712		
PLR	125.0 (90.9, 166.7)	0.5512		
Hemoglobin (Hb)	0.99 (0.98, 1.01)	0.5182		
Albumin (Alb)	0.95 (0.82, 1.10)	0.5181		
AFP	1.04 (0.89, 1.23)	0.6057		
CEA	1.00 (0.97, 1.03)	0.8507		
CA125	1.08 (1.01, 1.16)	**0.0098**	1.11 (1.02, 1.20)	**0.0123**
CA242	1.00 (1.00, 1.01)	0.4783		
CA199	1.00 (1.00, 1.00)	0.9661		
CA724	1.01 (0.98, 1.03)	0.4865		
Tumor location (%)	2.92 (0.91, 9.44)	**0.0426**	10.61 (1.34, 84.22)	0.254
Lauren classification (%)	0.21 (0.06, 0.75)	**0.0163**	0.40 (0.18, 0.88)	**0.0219**
CLDN18.2	0.86 (0.76, 0.97)	**0.0169**	0.86 (0.75, 0.99)	**0.0301**
Tumor differentiation type (%)	0.12 (0.01, 1.11)	**0.0416**	0.03 (0.00, 2.17)	0.1096

^*^Refers to the clinical stage of the cancer (cTNM)

*NLR* neutrophil-to-lymphocyte ratio, *PLR* platelet-to-lymphocyte ratio, *CLDN18.2* Claudin18.2 in Gastric Endoscopic Biopsy Specimen Immunohistochemistry Score

*Note*: Boldface indicates statistical significance (*P* <0.05)

**Fig. 4:**
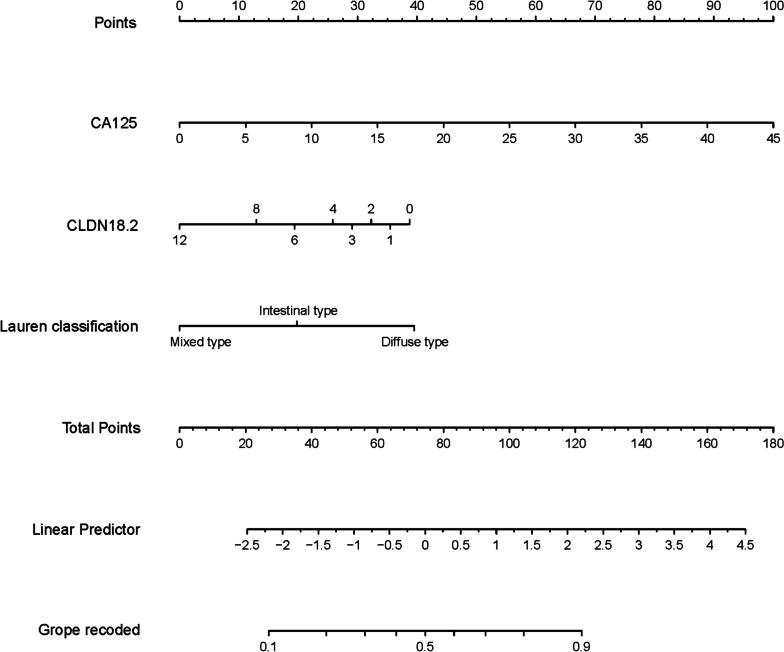
Nomogram for predicting occult peritoneal metastasis in progressive gastric cancer

Utilizing logistic regression analysis, a predictive nomogram model for occult peritoneal metastasis in gastric cancer was constructed using R software (shown in Fig. 4).

### Performance of the nomogram

**Fig. 5:**
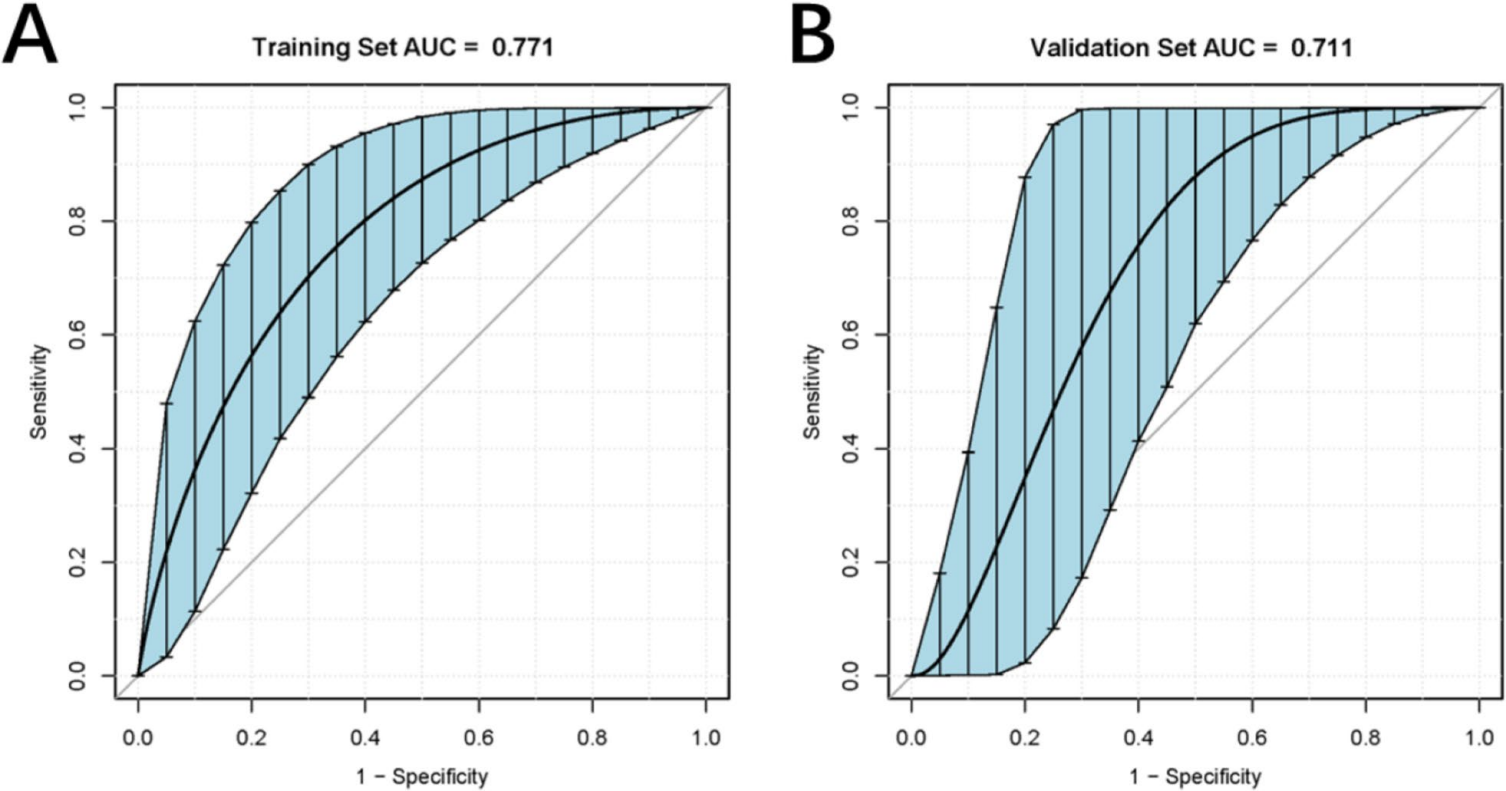
Receiver operating characteristic (ROC) curves predicting occult peritoneal metastases. **A** The training set. **B** The validation set. AUC, area under the receiver operating characteristic curve

**Fig. 6:**
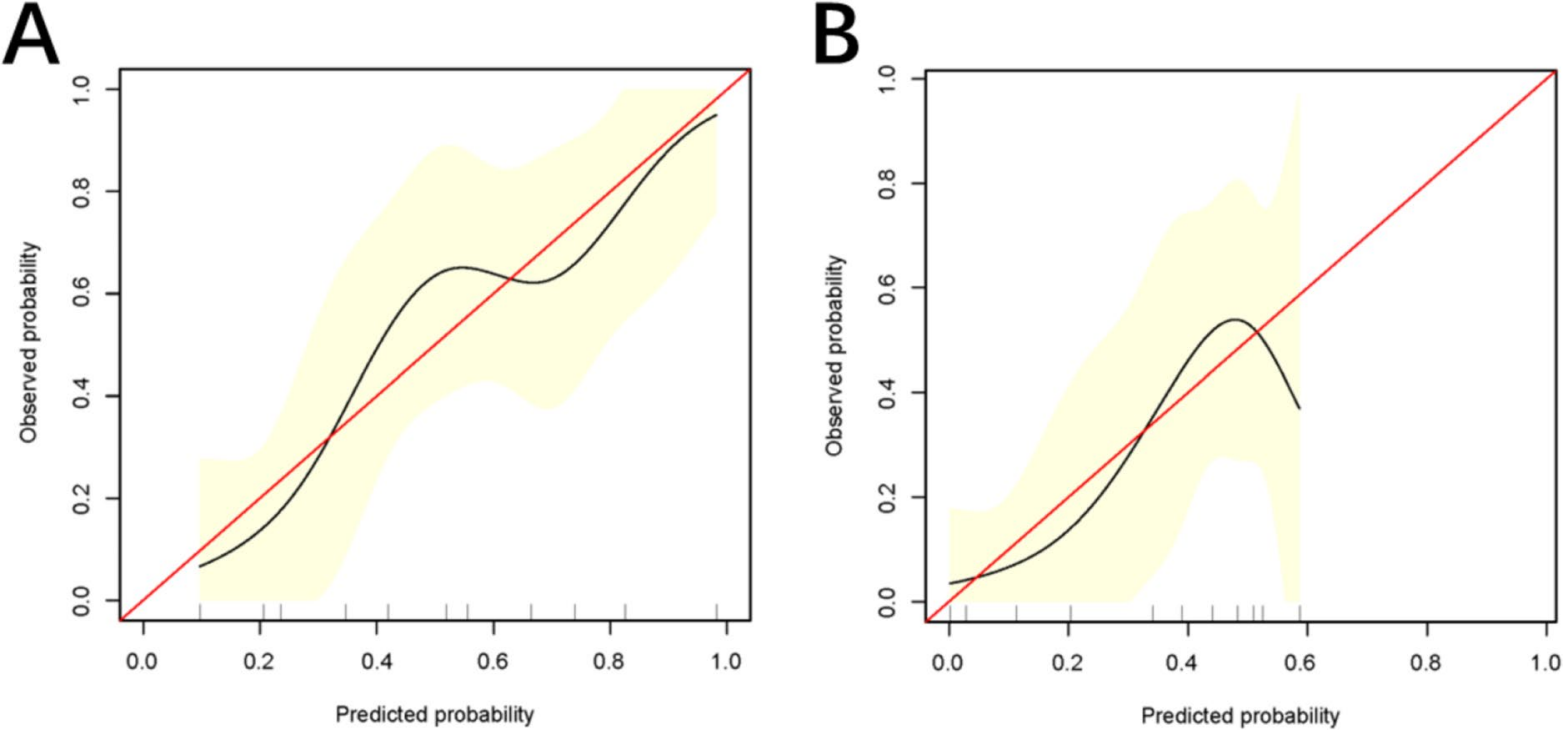
Evaluating nomogram model for occult peritoneal metastasis by calibration curve analysis. **A** The training set. **B** The validation set

**Fig. 7:**
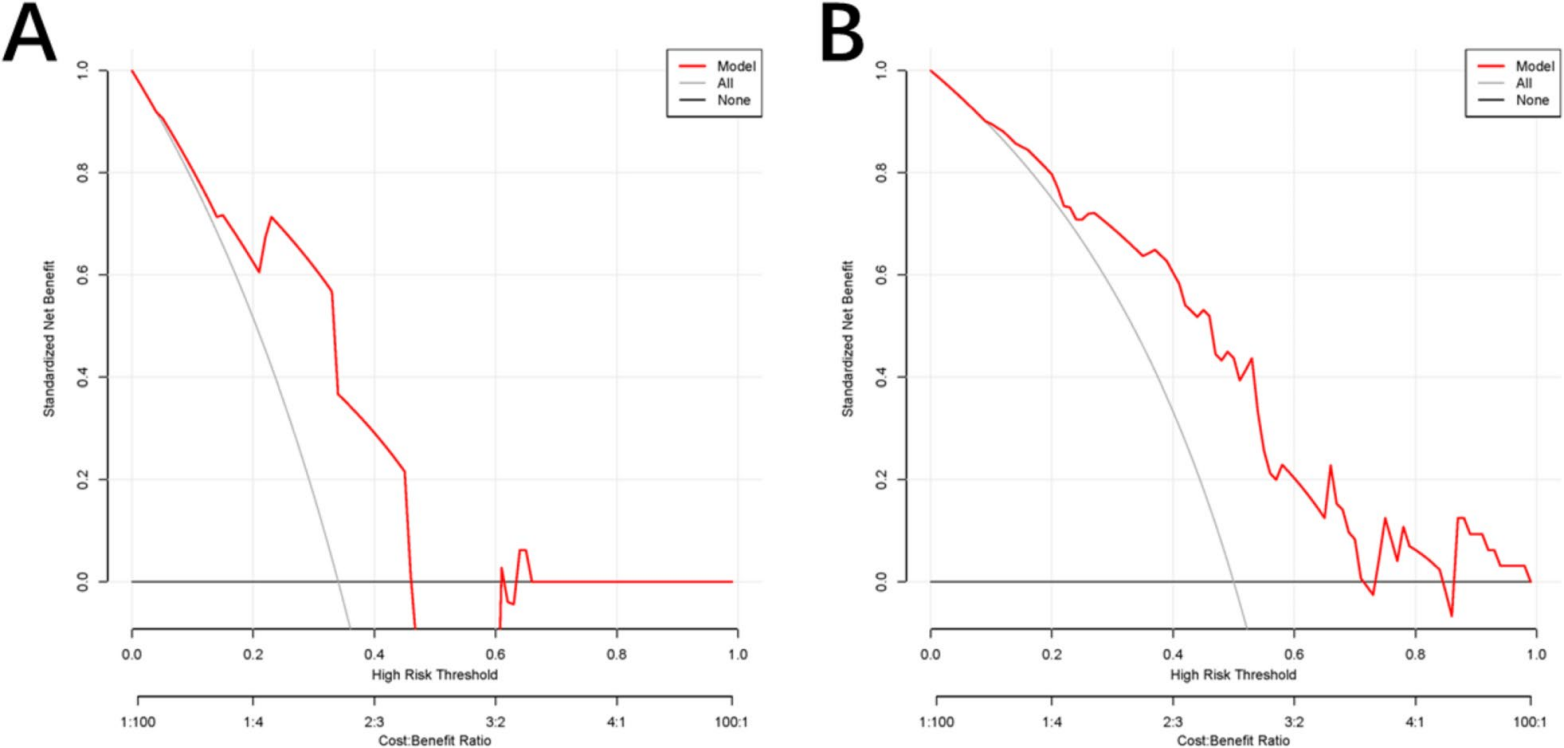
Decision curve analysis (DCA) to evaluate our nomogram. **A** The training set. **B** The validation set

The ROC curve of the nomogram model was subjected to 1000 repetitions of bootstrap resampling, yielding an area under the curve (AUC) of 0.771. In the validation group, the AUC was determined to be 0.711 (Fig. 5A, B). The calibration curve demonstrated that the predicted probabilities from the model fluctuated around the ideal curve, indicating good consistency (Fig. 6A, B). The goodness of fit of the nomogram model was evaluated using the Hosmer–Lemeshow test in SPSS software. The results showed that the model’s fit was consistent with the ideal curve, indicating a good fit for the nomogram model established in this study. Furthermore, the decision curve analysis (DCA) curve was plotted to assess the clinical utility of the nomogram model. The DCA curve revealed a larger area under the curve compared to single-factor analysis, indicating a higher clinical net benefit associated with the nomogram model (Fig. 7A, B).

## Discussion

The results of this study revealed a significant association between tumor location (*P* = 0.0426), Lauren classification (*P* = 0.0163), tumor differentiation type (*P* = 0.0416), serum CA125 levels (*P* = 0.0098), and CLDN18.2 score (*P* = 0.0169) with the occurrence of occult peritoneal metastasis (OPM) in gastric cancer. On the other hand, no significant relationship was found between AFP, CEA, CA242, CA199, CA724, neutrophil count (N), lymphocyte count (L), platelet count (P), neutrophil-to-lymphocyte ratio (NLR), platelet-to-lymphocyte ratio (PLR), hemoglobin (Hb), albumin (Alb), and the occurrence of OPM. Multivariate analysis demonstrated that Lauren classification, CA125, and CLDN18.2 score were independent risk factors for the development of occult peritoneal metastasis in gastric cancer.

CA125 is a glycoprotein expressed on the surface of mesothelial cells lining the body cavities. It has been widely used as a tumor marker due to its high sensitivity and specificity in the diagnosis of various gastrointestinal cancers. Studies by Nakata et al. have reported a close association between elevated serum CA125 levels and peritoneal dissemination, suggesting a poor prognosis in gastric cancer. Serum CA125 levels have been proposed as an alternative biomarker for predicting peritoneal metastasis and prognosis [16]. In one study, CA125 levels were found to be associated with survival [17]. Consistent with previous research, our findings in over 100 gastric cancer patients showed that the detection of CA125 levels can reflect the status of peritoneal involvement in gastric cancer. We observed a significant correlation between CA125 and peritoneal metastasis and identified it as an independent risk factor for peritoneal metastasis in the multivariate analysis. This finding suggests that CA125 may have biological significance in the progression or mitigation of peritoneal involvement in gastric cancer. In fact, previous studies have indicated that CA125 on the surface of cancer cells interacts with the mesothelin molecule on the surface of mesothelial cells, promoting cell adhesion to mesothelial cells and facilitating the formation of peritoneal metastasis [18, 19]. Therefore, elevated CA125 levels may not only be a consequence of disease progression but may also have a causal relationship with the progression of peritoneal metastasis.

In a study conducted by Jun H. Lee et al. [20], it was found that diffuse-type gastric cancer had a higher incidence of peritoneal metastasis compared to intestinal-type gastric cancer, with rates of 37% and 15%, respectively, showing a significant difference. Additionally, patients with diffuse-type and mixed-type gastric cancer had poorer prognosis after recurrence compared to patients with intestinal-type gastric cancer. This may be attributed to the following factors: firstly, diffuse-type gastric cancer exhibits higher invasiveness than intestinal-type gastric cancer, which potentially gives it a greater ability to migrate beyond the primary tumor site. Secondly, the cells of diffuse-type gastric cancer possess a higher capability of transitioning from epithelial cells to mesenchymal cells, further promoting the possibility of peritoneal metastasis [21]. Thirdly, the cells of diffuse-type gastric cancer may have a greater propensity to interact with the microenvironment present on the peritoneal surface, leading to the formation of metastatic deposits and subsequent implantation on the peritoneal surface.

In this study, it was observed that patients with occult peritoneal metastasis had significantly decreased expression of CLDN18.2 compared to those without peritoneal metastasis (mean IRS score 2 vs. 6, *P* = 0.013), indicating a correlation between loss of CLDN18.2 expression and the process of peritoneal metastasis in gastric cancer. The study by Seo Ree Kim et al. [13] revealed that out of 77 patients, 64.9% (50 cases) exhibited peritoneal metastasis. Interestingly, patients with peritoneal metastasis showed significantly lower CLDN18.2 expression compared to those without, aligning with our study’s findings. Limited clinical research currently exists on CLDN18.2 expression in gastric cancer peritoneal metastasis. In a study involving 74 gastric cancer patients, Tadayuki Oshima et al. [22] found that CLDN18.2 levels were inversely proportional to the Ki-67 labeling index at the invasive front of gastric cancer. They further demonstrated that transfecting MKN74 gastric cancer cells with CLDN18.2 siRNA enhanced their invasive abilities. Research by Susan J. Hagen et al. [23] indicated that the absence of the tight junction protein Claudin-18 accelerates mouse gastric cancer progression. Together, these findings suggest that reduced CLDN18.2 expression might play a crucial role in the progression and metastasis of gastric cancer.

CLDN18.2, a member of the Claudin family, is a crucial component of tight junctions involved in regulating cell–cell barrier function. The CLDN18.2 protein participates in tumor cell proliferation, differentiation, and migration. Due to its specific expression pattern, CLDN18.2 has emerged as a unique molecular target for targeted therapy in various cancers, particularly in gastric cancer. Different therapeutic agents targeting CLDN18.2 have been developed for cancer immunotherapy. Zolbetuximab is a novel chimeric immunoglobulin G1 antibody that specifically targets and binds with high affinity to the CLDN18.2 protein through its first extracellular domain, demonstrating no cross-reactivity with other Claudin family members [24, 25]. Clinical studies have verified the enhanced clinical benefits and safety profile of zolbetuximab in combination with other chemotherapeutic agents for patients with gastric cancer exhibiting high CLDN18.2 expression (moderate-to-strong expression in ≥ 75% of tumor cells). However, there has not been notable improvement in progression-free survival (PFS) and overall survival (OS) in patients with moderate to high expression (40–69% tumor cell range) [26]. The SPOTLIGHT trial, a global double-blind phase III study designed to assess the efficacy of modified FOLFOX6 (mFOLFOX6) combined with zolbetuximab versus mFOLFOX6 with placebo in CLDN18.2-positive advanced gastric cancer patients, revealed that only 38% (922 out of 2403) of patients reached the critical threshold for CLDN18.2 positivity [27]. These findings highlight the need to tailor treatment approaches for gastric cancer patients based on their CLDN18.2 expression levels. Furthermore, exploring mechanisms underlying gastric cancer patients with low CLDN18.2 expression is essential for devising targeted therapies and combined treatment strategies involving conventional radiotherapy, chemotherapy, and others.

Currently, the CLDN18.2 immunohistochemical examination has been incorporated into the routine postoperative pathological evaluation of gastric cancer patients at our research center. It is believed that the inclusion of this examination will lead to a more rational formulation of treatment plans for CLDN18.2-positive patients and result in improved treatment outcomes.

Gastric cancer patients with peritoneal metastasis often have a poor prognosis, and those with occult peritoneal metastasis are more prone to being overlooked, leading to missed opportunities for optimal treatment. Therefore, this study aimed to investigate the main clinical characteristics of occult peritoneal metastasis in gastric cancer patients and summarize the associated risk factors for its occurrence. These findings can serve as a clinical diagnostic basis for identifying patients with occult peritoneal metastasis in gastric cancer, thereby facilitating early diagnosis and treatment initiation to improve patients’ overall survival. However, it is important to note that this study had limitations, including a limited sample size and being conducted at a single center. Therefore, further data analysis involving larger sample sizes and prospective multiple centers is required to elucidate more accurate risk factors.

## Conclusion

In summary, our investigation has identified diffuse-type Lauren classification, elevated CA125 levels, and reduced CLDN18.2 expression as independent prognostic factors associated with occult peritoneal metastasis in gastric cancer patients. We have developed a meticulously designed nomogram to accurately predict the probability of occult peritoneal metastasis in advanced-stage gastric cancer patients, and its performance has been validated externally. Integration of these risk factors in clinical practice is crucial for vigilant surveillance of occult peritoneal metastasis in gastric cancer patients, ultimately leading to enhanced diagnostic precision and improved prognostic outcomes.

## Data Availability

The data are not publicly available due to privacy or ethical restrictions. Access to the data and the calculation method can be obtained from the corresponding author by email (wangmeng1980@nju.edu.cn).
